# Comparative Transcriptome Analysis Reveals Key Genes and Pathways Involved in Prickle Development in Eggplant

**DOI:** 10.3390/genes12030341

**Published:** 2021-02-25

**Authors:** Lei Zhang, Haoyun Sun, Tao Xu, Tianye Shi, Zongyun Li, Wenqian Hou

**Affiliations:** Jiangsu Key Laboratory of Phylogenomics and Comparative Genomics, School of Life Sciences, Jiangsu Normal University, Xuzhou 221116, China; leizhang@jsnu.edu.cn (L.Z.); 2020180495@jsnu.edu.cn (H.S.); xutao_yr@126.com (T.X.); 2020180490@jsnu.edu.cn (T.S.); zongyunli@jsnu.edu.cn (Z.L.)

**Keywords:** eggplant, prickle, transcriptome, development

## Abstract

Eggplant is one of the most important vegetables worldwide. Prickles on the leaves, stems and fruit calyxes of eggplant may cause difficulties during cultivation, harvesting and transportation, and therefore is an undesirable agronomic trait. However, limited knowledge about molecular mechanisms of prickle morphogenesis has hindered the genetic improvement of eggplant. In this study, we performed the phenotypic characterization and transcriptome analysis on prickly and prickleless eggplant genotypes to understand prickle development at the morphological and molecular levels. Morphological analysis revealed that eggplant prickles were multicellular, lignified and layered organs. Comparative transcriptome analysis identified key pathways and hub genes involved in the cell cycle as well as flavonoid biosynthetic, photosynthetic, and hormone metabolic processes during prickle development. Interestingly, genes associated with flavonoid biosynthesis were up-regulated in developing prickles, and genes associated with photosynthesis were down-regulated in developing and matured prickles. It was also noteworthy that several development-related transcription factors such as bHLH, C2H2, MYB, TCP and WRKY were specifically down- or up-regulated in developing prickles. Furthermore, four genes were found to be differentially expressed within the *Pl* locus interval. This study provides new insights into the regulatory molecular mechanisms underlying prickle morphogenesis in eggplant, and the genes identified might be exploited in breeding programs to develop prickleless eggplant cultivars.

## 1. Introduction

Eggplant (*Solanum melongena* L.) is cultivated and consumed all over the world, ranking as the third most important *Solanaceae* crop after potato and tomato [1]. Eggplant is the only Old World *Solanaceous* crop that has experienced a long period of domestication [2], and is also unique in possessing a prickly phenotype in *Solanaceous* crops [3,4]. Prickles can occur at the surface of the leaves, stems and fruit calyxes of eggplant, which could protect it against the attacks from herbivores and mechanical injury [5]. However, the prickles are considered undesirable because they may increase the difficulty of cultivation, harvesting and transportation [4]. The investigations of prickle characteristic variations in eggplant populations revealed that most of the eggplant cultivars (at least 63.1% of all assessed eggplants) have prickles on fruit calyx [6,7]. The development of prickleless eggplant cultivars is thus an important objective in breeding programs.

Previous studies on raspberry and blackberry prickles have shown that they are outgrowths of epidermal tissue generated from modified glandular trichomes (GTs); once the outermost cells become lignified, lignification continues inward and downwards until the prickles become completely lignified and thus mature [8,9]. A phenotypic analysis of the prickles in *Solanum viarum* Dunal revealed that they may initiate from GTs or be induced by some signals derived from GTs [10]. Transcriptome analyses in raspberry and *S. viarum* further identified several transcription factors (TFs) that may play important roles in prickle development [10,11]. In eggplant, several quantitative trait loci (QTLs) for prickle have been identified on chromosomes 2, 6, 7 and 8 [3,12,13,14]. A recent study has genetically mapped a *Pl* locus on chromosome 6, and developed a 0.5 kb presence/absence variant marker for the marker-assisted selection of prickleless eggplant [4]. However, our knowledge of the genetic regulatory networks underlying prickle morphogenesis in eggplant remains poor.

Similar as prickles, trichomes are also an outgrowth derived from the epidermis of plants; thus, studies of trichomes might help us understand the molecular basis of prickle morphogenesis in eggplant. Some key genes involved in trichome development have been identified. In *Arabidopsis*, the trichomes are unicellular, and their initiation and patterning are under the control of the WD-repeat/bHLH/MYB complex, which consists of three classes of TFs (including the R2R3–MYB protein GL1, bHLH protein GL3/EGL3 and WD40 repeat protein TTG1) [15]. In addition, the WD-repeat/bHLH/MYB complex functions directly with the HD-ZIP IV family protein GLABRA 2 (GL2) to initiate trichome formation [15]. In cotton, trichomes are generally referred to as fibers, which are also a type of single-cell organ. Many genes involved in fiber development in cotton, such as *GhMYB2* [16], *GhTTG1* [17], *GhDEL61/65* [18], and *GhHOX1/2/3* [19,20], can restore the trichome phenotype in corresponding *Arabidopsis* homolog mutants, indicating a similar regulatory network between cotton and *Arabidopsis*. Compared to the unicellular trichome, the formation of the multicellular trichome in other species engaged a different regulatory pathway [21,22]. In cucumber, the multicellular trichomes on fruit are also called fruit spines. Genetic and molecular analyses revealed that genes encoding HD-ZIP proteins, such as *CsGL1*, *CsGL3*, *TBH* and *TRIL*, are responsible for fruit trichome development [23]. In tomato, *Wo* (HD–ZIP protein) and *H* (single C2H2 zinc-finger protein) were identified via genetic mapping and were found to be functionally conserved in multicellular trichome formation in *Solanaceae* species [21,24]. In general, the molecular mechanisms underlying the trichome development have been well studied in the model plant *Arabidopsis* and other species. However, the molecular basis of prickle morphogenesis has been rarely studied by comparison.

Here, we performed the morphological studies to understand the cellular structures of eggplant prickles, and then conducted RNA sequencing on the prickly and prickleless eggplant genotypes to identify candidate genes responsible for the development of prickles in eggplant. Our study provides new insights into the regulatory molecular mechanisms underlying prickle morphogenesis in eggplant. The genes identified in our study could be used in breeding programs to develop prickleless eggplant cultivars.

## 2. Materials and Methods

### 2.1. Plant Materials

The prickleless eggplant “Xianqie 23” was bought from the Chinese seed market, and the prickly eggplant “PI 381159” was ordered from the Germplasm Resources Information Network (GRIN) website. Eggplant seeds of the prickly genotype (PI 381159) and the prickleless genotype (Xianqie 23) were sown directly into soil and grown in a greenhouse at 23–26 °C during the day and 20–22 °C at night. The developing prickles on flower calyx from prickly eggplant, the matured prickles on fruit calyx from prickly eggplant, and the epidermis from prickleless eggplant were assigned into three groups: developing prickles, matured prickles and prickleless epidermis. Prickles or epidermis from ten individuals were pooled for one biological replicate. Three such independent biological replicates were used for each group. All replicates were immediately frozen in liquid nitrogen and stored at −80 °C until further processing.

### 2.2. Morphological Characterization and Microscopic Investigation

To get a three-dimensional view of the sample, stems from prickleless and prickly plants were observed by a stereo microscope (Leica S8 APO, Leica Microsystems, Wetzlar, Germany) and were photographed with a digital camera (Leica DMC4500, Leica Microsystems, Wetzlar, Germany) via the Leica Application Suite V4.10 software (Leica Microsystems, Wetzlar, Germany).

To study the structure and lignification of prickles, paraffin embedding and sectioning methods described below were used: (i) prickles from prickly plants were cut into an appropriate size (~1 cm); (ii) the sections were then rehydrated in xylenes (twice, each for 5 min), 100% alcohol (twice, each for 5 min) and 75% alcohol (twice, each for 5 min) and were flushed with running water; (iii) the sections were stained in safranin O staining solution for 2 h and washed with water; (iv) the sections were decolorized by 50%, 70% and 80% alcohol for 3–8 s each; (v) the sections were stained in plant solid green staining solution for 6–20 s and dehydrated with 100% ethanol for fast green staining; (vi) the sections were placed in xylene for 5 min and sealed with neutral balsam; (vii) the sections were photographed by Nikon Eclipse E100 microscope (Nikon, Tokyo, Japan) with a Nikon DS–U3 digital camera (Nikon, Tokyo, Japan).

To further analyze the cell morphology of prickles, scanning electron microscopy methods were used. The sections were first fixed in electron microscopy fixative for 2 h at room temperature and then washed three times (15 min each) with phosphate buffered saline (PBS) solution. Next, the sections were post-fixed in 1% OsO_4_ for 2 h at room temperature, washed with fresh PBS solution, and then dehydrated by 30%, 50%, 70%, 80%, 90%, 95% and 100% (twice) ethanol and isoamyl acetate for 15 min. After the sections were dried by a Critical Point Dryer (K850, Quorum Emitech, Ashford, Kent, UK), they were attached to aluminum stubs and coated with gold palladium. Images were taken with a scanning electron microscope (SU810, Hitachi, Tokyo, Japan).

### 2.3. RNA Isolation and Illumina Sequencing

Total RNAs of nine samples (three biological replicates for each of the three groups, which were prickleless epidermis, developing prickles, and matured prickles) were extracted using TRIzol™ Reagent (Invitrogen, Carlsbad, CA, USA) according to manufacturers’ instructions. RNA quantity and quality were assessed by agarose gel, spectrophotometric analysis (ND1000, NanoDrop Technologies, Wilmington, DE, USA), and an Agilent 2100 Bioanalyzer (Agilent Technologies, Santa Clara, CA, USA). The non-stranded paired-end cDNA library of each sample was constructed by the Illumina TruSeq RNA Sample Prep Kit v2 (Illumina, San Diego, CA, USA). The libraries were sequenced on the Illumina NovaSeq 6000 platform to generate 150 bp paired-end reads.

### 2.4. Transcriptome Sequence Processing and Analysis

Sequencing data analysis was performed according to our previous studies [25,26]. High-quality clean reads for each sample were obtained from raw RNA-seq data by removing adaptor sequences and ambiguous nucleotides using Trimmomatic (version 0.34) [27] and then were mapped to the eggplant genome HQ–1315 [28] using STAR (version 2.7.1a) under the 2-pass mapping mode [29]. A total of nine BAM files were generated and used in subsequent analyses.

The read numbers mapped to each gene were counted by featureCounts (version 2.0.1) [30], and the TPM (transcripts per million) of each gene was calculated using an in-house Perl script. The DEseq2 package [31] was used to perform differential gene expression analysis between groups (three biological replicates per group). The *p*-value for each gene was adjusted using the Benjamini and Hochberg’s method [32]. Genes with the adjusted *p*-values < 0.001 and absolute log_2_ fold changes (log_2_FC) > 2 were considered as differentially expressed genes (DEGs). DEGs with similar expression profiles were further partitioned into clusters using the k-means clustering algorithm implemented in Trinity (version 2.1.1) [33].

### 2.5. Functional Annotation and GO Enrichment Analysis

The genome-wide functional annotation of eggplant genes was assigned using the eggNOG-mapper pipeline (version 2) [34] with default parameters. The OrgDb database for eggplant was built using an in-house R script. Gene Ontology (GO) enrichment analysis of DEGs was conducted using clusterProfiler [35]. GO terms with adjusted *p*-values < 0.05 were considered significantly enriched.

### 2.6. Expression Level Validation of DEGs by qRT-PCR

First-strand cDNA was synthesized from 1.0 μg of total pure RNA using the HiFiScript cDNA Synthesis Kit (CoWin Biosciences, Beijing, China). Gene-specific primers for the selected DEGs were designed using Primer3Plus [36]. qRT-PCR was performed on a CFX96 real-time PCR System (Bio-RAD, Hercules, CA, USA) using SYBR^®^ Premix Ex Taq^TM^ II (TaKaRa, Shiga, Japan). *SmAPRT* was used as a reference gene to normalize the expression levels [37]. The relative expression levels for each gene were quantified using the 2^–ΔΔCt^ method [38]. All primers are listed in Supplemental Table S12.

## 3. Results

### 3.1. Morphological Characterization and Microstructure of Prickly and Prickleless Eggplants

**Figure 1:**
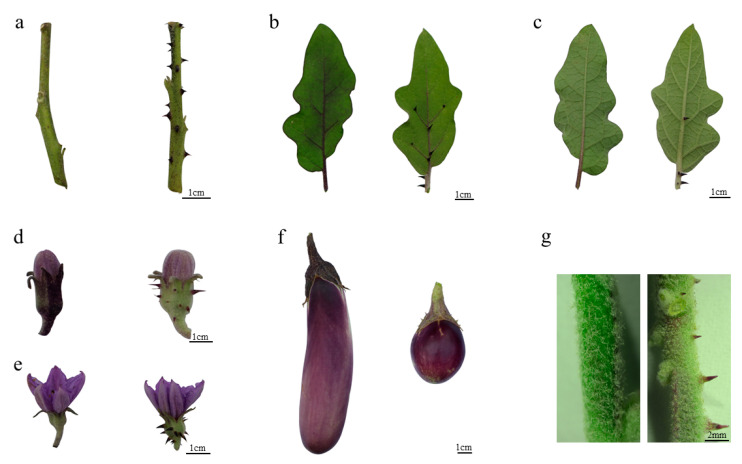
Morphological comparison of prickleless and prickly eggplants. (**a**) Stems from prickleless and prickly eggplants; (**b**) Adaxial side of leaves from prickleless and prickly eggplants; (**c**) Abaxial side of leaves from prickleless and prickly eggplants; (**d**) Budding flowers from prickleless and prickly eggplants; (**e**) Blooming flowers from prickleless and prickly eggplants; (**f**) Fruits from prickleless and prickly eggplants; (**g**) Stereo microscope images of stems from prickleless and prickly eggplants. Black bars are scales of either 1 cm or 2 mm.

The most significant phenotypic difference between the prickly genotype (PI 381159) and the prickleless genotype (Xianqie 23) of eggplant was the presence of prickles on the aboveground organs (Figure 1a–f). Developing and matured prickles were observed on the stems, leaves and calyxes of the flower and fruit in the prickly genotype. Conversely, no prickles were detected on these same organs in the prickleless genotype. The epidermis of the stem was green in color, and the prickles were purple. Observations of the stem with a stereo microscope revealed that the surfaces of the prickly and prickleless genotype were all covered with a dense layer of trichomes (Figure 1g). The prickles of eggplant were also much sharper, harder and larger than trichomes (Figure 1g).

**Figure 2:**
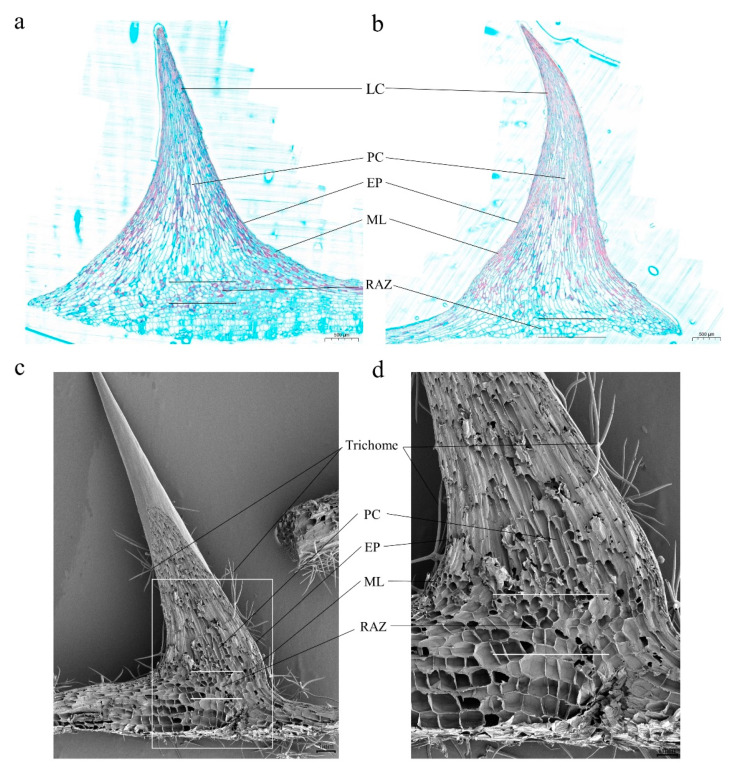
Micrographs of the structures of prickles. (**a**) Semi-thin section and Safranin O/Fast Green staining of longitudinally sliced developing prickles; (**b**) Semi-thin section and Safranin O/Fast Green staining of longitudinally sliced matured prickles; (**c**) Scanning electron micrographs of longitudinally sliced matured prickles; (**d**) Enlarged view of the area outlined by the white square in the graph (**c**). LC, Lignified Cell; EP, Epidermis; ML, Meristematic Layer, PC, Parenchyma Cell; RAZ, Resembling Abscission Zone. Bars, 500 µm in (**a**) and (**b**), 3 mm in (**c**), 1 mm in (**d**).

Light and scanning electron microscopy were further used to characterize the cellular structure of developing and matured prickles on stems. Similar to trichomes, prickles of eggplant were also composed of multiple cells (Figure 2, Supplementary Figure S1). However, the basal area of prickles was larger than that of trichomes (Supplementary Figure S1). To study the lignification patterns during the development of prickles, we sectioned and stained the developing and matured prickles (Figure 2a,b). It is revealed that the lignification initially occurred in the outermost cells of prickles, and later continued in inward and downwards cells of prickles. We also found that the matured prickles had a higher degree of lignification than developing prickles. The developing and matured prickles can be divided into several parts (Figure 2). The outmost layer of prickles was the epidermis (EP), in which the cells were smaller, densely arranged, and square in shape. Below the EP was the meristematic layer (ML), which was composed of one or two layers of small and orderly arranged cells. The innermost layer of the prickles was made up of large, thin-walled, and loosely arranged parenchyma cells (PC). The basal area of prickles resembled an abscission zone (RAZ) that included three to five layers of small, round, closely and horizontally arranged cells.

### 3.2. Transcriptome Sequencing and Read Mapping

To gain insights into the molecular mechanisms underlying prickle development, a total of nine cDNA libraries on developing prickles, matured prickles and prickleless epidermis were constructed for transcriptome sequencing. Utilizing the Illumina NovaSeq 6000 platform, a total of 66.68 Gb (444.54 million paired-end reads) raw data were generated. After removing low quality reads, approximately 62.58 Gb (418.03 million paired-end reads) clean data were obtained, ranging from 6.29 to 7.67 Gb per library (Supplementary Table S1).

The clean data of each sample were then mapped to the eggplant genome HQ–1315 with an average mapping rate of 99.14% (Supplementary Table S1). The read counts and TPM values for all genes were calculated. In total, 21,273 expressed genes were detected from the transcriptome dataset, accounting for 58.17% of all annotated genes in the eggplant genome. The expressed genes were further used to generate a correlation matrix to compare the similarity of all transcriptomes. The heatmap revealed that three groups (developing prickles, matured prickles and prickleless epidermis) were distinguished, and the three biological replicates of each group were highly correlated (Supplementary Figure S2).

### 3.3. Analysis of DEGs

**Figure 3:**
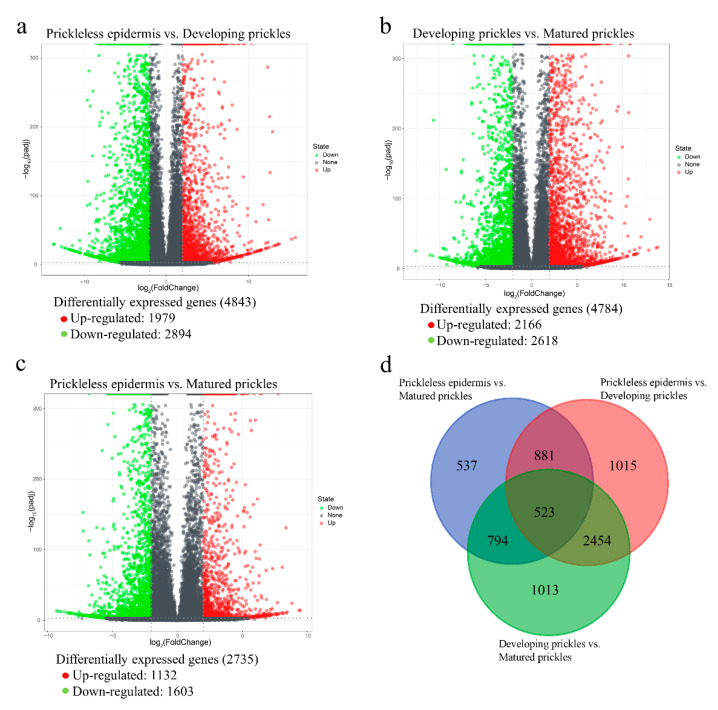
Volcano plots and Venn diagram of DEGs in the pairwise comparisons. (**a**) Volcano plot of DEGs in the prickleless epidermis vs. developing prickles comparison; (**b**) Volcano plot of DEGs in the prickleless epidermis vs. matured prickles comparison; (**c**) Volcano plot of DEGs in the developing prickles vs. matured prickles comparison; (**d**) Venn diagram of the number of DEGs in the pairwise comparisons.

To identify genes involved in the prickle development, a genome-wide expression analysis was performed for the prickleless epidermis, developing prickles, and matured prickles. Genes with adjusted *p*-values < 0.001 and absolute log_2_FC > 2 were considered as significantly DEGs (Supplementary Table S2–S4) and are shown in volcano plots (Figure 3a–c). Comparative transcriptome analysis revealed that the prickleless epidermis vs. matured prickles comparison generated the smallest number of DEGs (2735), followed by developing prickles vs. matured prickles comparison (4784), and prickleless epidermis vs. developing prickles comparison (4843). More specifically, a total of 4843 genes were differentially expressed in the prickleless epidermis vs. developing prickles comparison, including 1979 up-regulated genes and 2894 down-regulated genes. A total of 4784 DEGs were identified in the developing prickles vs. matured prickles comparison, and 2166 DEGs were up-regulated and 2618 DEGs were down-regulated. In the prickleless epidermis vs. matured prickles comparison, there were 2735 DEGs, of which 1132 were up-regulated and 1603 were down-regulated.

To explore the distribution of DEGs among pairwise comparisons, a Venn diagram was constructed (Figure 3d). Of all DEGs, a total of 523 genes were found to be differentially expressed in all comparisons. A total of 1015, 537, and 1013 genes were exclusively differentially expressed in the prickleless epidermis vs. developing prickles, prickleless epidermis vs. matured prickles and developing prickles vs. matured prickles comparisons during prickle development comparisons, respectively.

### 3.4. Functional Annotation of DEGs

**Figure 4:**
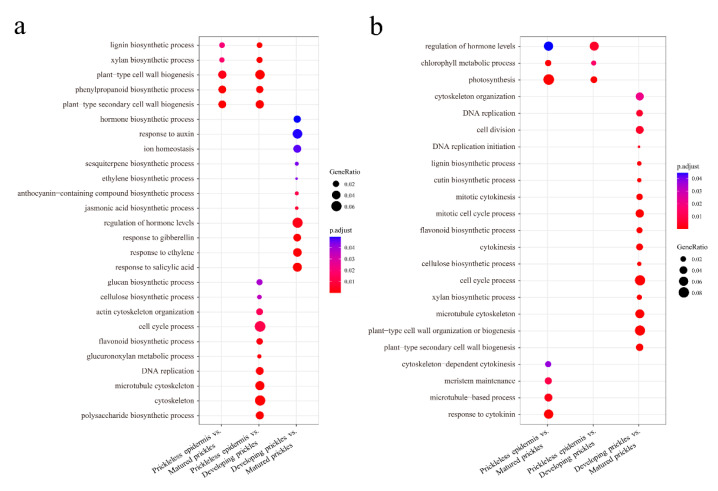
GO enrichment analysis of DEGs identified in the pairwise comparisons. (**a**) GO enrichment analysis of up-regulated DEGs identified in the pairwise comparisons. (**b**) GO enrichment analysis of down-regulated DEGs identified in the pairwise comparisons.

All of the annotated genes in eggplant were used as background and GO enrichment analysis was performed to characterize the biological functions of DEGs (Supplementary Table S5, Supplementary Figure S3). For example, GO terms related to “plant-type secondary cell wall biogenesis” and “phenylpropanoid biosynthetic process” were enriched in all comparisons. GO terms related to “photosynthesis” were enriched in the prickleless epidermis vs. developing prickles and prickleless epidermis vs. matured prickles comparisons. To better understand the biological functions of DEGs, enriched GO terms were obtained for the up- and down-regulated DEGs (Supplementary Tables S6 and S7, Figure 4). For instance, compared with prickleless epidermis, up-regulated DEGs in developing and matured prickles were significantly enriched in GO terms related to “plant-type cell wall biogenesis” and “phenylpropanoid biosynthetic process” (Figure 4a). This result was consistent with the characteristics of the prickles, which are hard and purple during the development. Down-regulated DEGs in developing and matured prickles were mainly overrepresented with GO terms related to “photosynthesis” (Figure 4b), which suggests that the photosynthetic activity in prickles was very low. We also found that GO terms related to “cell cycle process” were all significantly enriched in the up-regulated and down-regulated DEGs in developing prickles relative to prickleless epidermis and matured prickles, respectively (Figure 4). This result indicated that the cells in developing prickles are rapidly proliferating.

### 3.5. Expression Patterns of DEGs

**Figure 5:**
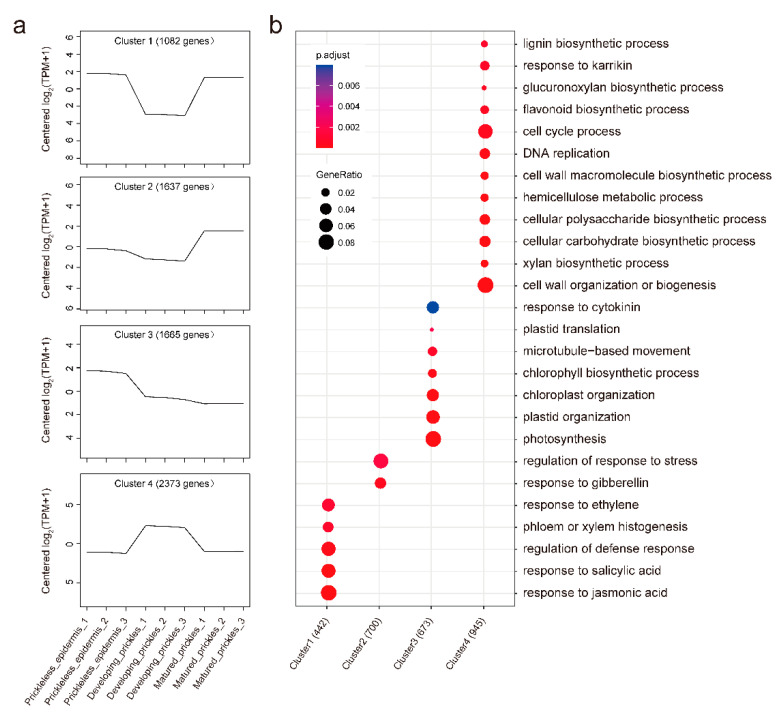
Expression patterns of DEGs during prickle development. (**a**) K-means clustering grouped DEGs into four clusters. (**b**) GO enrichment analysis among the four clusters using the clusterProfiler.

To investigate the expression patterns of DEGs during prickle development, clustering analysis was performed using the K-means clustering algorithm. A total of four clusters were obtained, which could be classified into four categories: down then up regulated (Cluster 1), up-regulated (Cluster 2), down-regulated (Cluster 3), and up- then down regulated (Clusters 4) (Figure 5a, Supplementary Table S8). GO enrichment analysis of genes in Clusters 3 and 4 uncovers more biologically meaningful results than those in Clusters 1 and 2. The expression of genes in Cluster 3 exhibited a general downward trend during the prickle development. GO terms related to photosynthesis were overrepresented in this cluster, such as “photosynthesis”, “chloroplast organization” and “chlorophyll biosynthetic process” (Figure 5b), which suggested that photosynthetic activity during the development of prickles was reduced. Contrary to the expression pattern of Cluster 3, the expression of genes in Cluster 4 rapidly increased in development prickles and then strongly decreased in mature prickles. Some GO terms related to flavonoid biosynthesis and cell cycle, such as “DNA replication,” and to cell wall biogenesis, such as “xylan biosynthetic process” and “lignin biosynthetic process,” were enriched (Figure 5b). These findings, along with the morphological observations, suggest that the genes in Cluster 4 play a key role in prickle growth.

### 3.6. DEGs Related to Cytoskeleton, DNA Replication and Cell Wall Biosynthesis

The cytoskeleton consists of microfilaments and microtubules is essential for cell morphogenesis [39]. Compared with prickleless epidermis, up-regulated DEGs in developing prickles were enriched in GO terms related to “cytoskeleton” and “microtubule motor activity” (Figure 4a, Supplementary Table S6a). For example, the expression levels of genes encoding microtubules associated proteins (MAPs), kinesin-like proteins (KLPs) and end binding proteins (e.g., EB1) were higher in developing prickles than in prickleless epidermis and matured prickles (Supplementary Figure S4a, Supplementary Table S9a). 

The process of DNA replication is a fundamental aspect of cell division [40]. GO enrichment analysis of DEGs revealed that DNA replication was overrepresented (Figure 4). We found that genes encoding key proteins related to DNA replication were highly expressed in developing prickles compared with prickleless epidermis and matured prickles, such as minichromosome maintenance complex proteins (MCM), origin recognition complex proteins (ORC), cell division control proteins (e.g., CDC6), cyclin and DNA helicases (Supplementary Figure S4b, Supplementary Table S9b).

The walls surrounding the cells provide mechanical support for plants. Functional analysis of DEGs revealed that GO terms related to “secondary cell wall biogenesis” were significantly overrepresented (Figure 4). Genes related to secondary wall biosynthesis in prickle cells were further analyzed, including six genes for cellulose biosynthesis and assembly, ten genes for xylan biosynthesis, ten genes for lignin biosynthesis and polymerization and nine genes encoding TFs involved in the secondary wall [41] (Supplementary Figure S4c; Supplementary Table S9c). Expression analysis revealed that these genes were highly expressed in developing prickles, followed by matured prickles and prickleless epidermis (Supplementary Figure S4c).

### 3.7. DEGs Related to Flavonoid Biosynthetic and Photosynthenic Processes

Compared with prickleless epidermis, GO analysis revealed that “flavonoid biosynthetic process” was significantly enriched in up-regulated DEGs (Figure 4a) and “photosynthesis” was significantly overrepresented in down-regulated DEGs in developing and matured prickles (Figure 4b). To determine the key genes involved in these two processes, the expression of all genes involved were further explored. We found that ten genes encoding key enzymes or regulatory factors in flavonoid biosynthetic process [26,42] were highly expressed in prickles, including flavanone 3-hydroxylase (F3H), chalcone synthase (CHS), chalcone isomerase (CHI), O-methyltransferase 1 (OMT1), and MYB domain protein (MYB12/MYB111) (Supplementary Figure S4d, Supplementary Table S9d). On the contrary, genes involved in photosynthesis such as photosystem I, photosystem II, light harvesting and chlorophyll biosynthesis were expressed at low levels in developing and matured prickles (Supplementary Figure S4e, Supplementary Table S9e).

### 3.8. TFs Related to Prickle Development

TFs have been reported to play essential roles in the development of plant organs. In our study, trichome development-related genes in *Arabidopsis* were used to explore candidate TFs related to prickle development in eggplant. A total of 12 TFs from eight families were identified to be differentially expressed during the prickle development, including bHLH, C2H2, MYB, TCP and WRKY (Supplementary Figure S5, Supplementary Table S10). Cluster analysis divided these genes into two groups: up-regulated genes in developing prickles and down-regulated genes in developing prickles.

### 3.9. Identification and Verification of the DEGs Located in the Pl Locus

**Figure 6:**
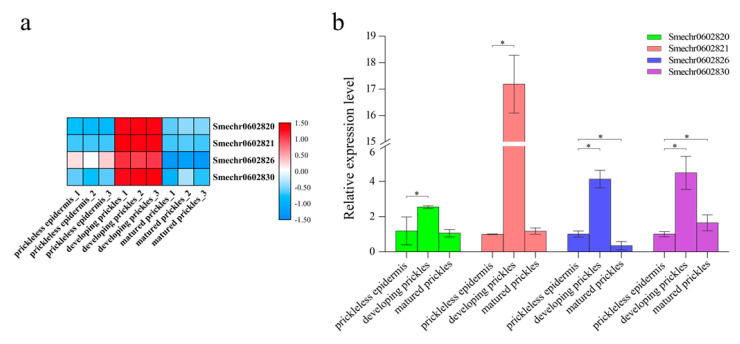
Heatmap analysis and qRT-PCR validation of the DEGs within the *Pl* locus interval. (**a**) Heatmap showing the expression patterns of the DEGs within the *Pl* locus interval. The heatmap was generated based on the normalized log_2_(TPM+1) values for each DEG. Blue and red scales represent relatively low or high expression, respectively. (**b**) qRT-PCR validation of the DEGs within the *Pl* locus interval. The results were expressed as mean ± SD, * for *p*-value < 0.05.

A recent genetic study has identified a *Pl* locus that can lead to the absence of prickles in eggplant, which is located on chromosome 6 with an interval of 133 kb [4]. Using the eggplant genome HQ–1315 as the reference, a total of 14 candidate genes were annotated, including alpha carbonic anhydrase, nudix hydrolase, glycosyltransferase and GATA TF etc. (Supplementary Table S11). The transcriptome data in our study were further used to analyze these candidate genes, and 11 genes were expressed (Supplementary Figure S6). Of these genes, four genes were significantly differentially expressed, which encoded nudix hydrolase homolog (Smechr0602820), lysine decarboxylase (Smechr0602821), auxin response factor (Smechr0602826) and galactosyltransferase (Smechr0602830) (Figure 6a). qRT-PCR analysis was then performed to verify the differential expression of these four genes. Generally, the expression patterns determined by qRT-PCR were consistent with those obtained by RNA-seq (Figure 6b), which confirmed the reliability and accuracy of our transcriptome data.

## 4. Discussion

Compared with the wild relatives of eggplant, morphological features of cultivated eggplant have greatly changed, such as large, palatable fruit and fewer prickles [43]. Prickliness is an important quality trait in eggplant, as the prickly eggplant is not convenience for humans to cultivate, harvest, and transport [12]. The development of prickleless eggplant cultivars is thus an important goal of breeding programs. However, a lack of knowledge of the molecular mechanisms underlying the prickle morphogenesis has hindered the exploitation of genetic engineering technologies to accelerate eggplant genetic improvement. In this study, we performed the phenotypic characterization and transcriptome analysis on prickly and prickleless eggplant genotypes to understand prickle development at the morphological and molecular levels.

### 4.1. Multicellular, Lignified, and Layered Eggplant Prickles

Morphological studies of prickles on raspberry, blackberry, and *S. viarum* have shown that prickles may either been generated from GTs or induced by the signals derived from GTs; in addition, prickles were shown to be multicellular and lignified, and could be divided into several parts [9,10]. In our study, morphological analysis revealed that prickles were composed of multiple cells, and the lignification level increased during growth. Similar to prickles from other species, eggplant prickle cells exist in different shapes and sizes, and they could roughly be divided into different parts, including EP, ML, PC and RAZ (Figure 2). However, we could not find any GTs on the epidermis of the stem, indicating that eggplant prickles may not be derived from GTs; thus, the molecular mechanisms underlying the morphogenesis of eggplant prickles may be different from those of GT derived prickles.

### 4.2. Key Genes and Pathways Involved in Prickle Development

The availability of a high-quality eggplant genome serves as a basis for omics projects [28]. Our study showed that the morphogenesis of prickles is an asynchronous process, and prickles at different stages of development could be observed in the same organ. Therefore, we assumed that the development of prickles did not show any tissue specificity in the aboveground organs. We speculated that the prickles on any one organ could represent the prickles on the other organs, and genes that are associated with the development of prickles on any organ may also be involved in the development of prickles on the other organs. To reveal the molecular mechanisms of prickle development, transcriptome sequencing of prickles on calyxes of flower and fruit from prickly eggplant and epidermis from prickleless eggplant was conducted in this study. A total of 21,273 expressed genes were detected using Illumina sequencing technology. Of these genes, 7217 candidate genes were differentially expressed during prickle development. Pairwise comparisons of transcriptomes revealed that the developing prickles possess the largest number of DEGs, indicating that developing prickles may experience more dramatic expression changes compared with prickleless epidermis and matured prickles. Four co-expressed clusters were grouped for DEGs using the K-means algorithm, suggesting that complex processes may underly prickle morphogenesis. Functional analysis of DEGs and co-expressed clusters revealed that GO terms related to “cell-cycle”, “flavonoid biosynthesis process” and “photosynthesis” were enriched, implying that they contribute to prickle development.

Plant growth and development are sustained by coordinated cellular behaviors, such as cell division, expansion, and differentiation. It is known that the cytoskeleton, DNA replication and cell wall biosynthesis are important in these cellular behaviors [40,44,45,46]. These processes are also essential in trichome morphogenesis [47,48,49]. Morphological analysis of eggplant prickles revealed that cells in developing prickles proliferate rapidly, and cells in matured prickles stop dividing and undergo lignification. This phenotype could be explained by our transcriptome analysis. Genes involved in the cytoskeleton, DNA replication, and cell wall biosynthesis were significantly highly expressed in developing prickles compared with prickleless epidermis and mature prickles, indicating that these processes are important for the development of eggplant prickles (Supplementary Figure S4a–c). Among these genes, the homolog of *AtKinesin-13A* (*Smechr0502712*), which is involved in trichome morphogenesis [50], was significantly up-regulated in developing prickles, suggesting that it may play a role in prickle development.

A key feature of prickles is the capacity to synthesize and store a large number of secondary metabolites, including flavonoids, terpenoids, and alkaloids [10]. In this study, phenotypic analysis revealed that eggplant prickles were purple during development (Figure 1g). Transcriptome analysis showed that ten genes associated with flavonoid biosynthesis were highly expressed in the developing prickles (Supplementary Figure S4d). Of these genes, *MYB12* (*Smechr0600048*) and *MYB111* (*Smechr0600051*) are the known master regulators that can strongly activate the promoters of downstream structural genes in plants [42]. Our study indicated that these two genes are responsible for flavonoid accumulation in eggplant prickles. By contrast, genes encoding key proteins related to chlorophyll biosynthesis were significantly down-regulated in both types of prickles, especially in matured prickles, such as GUN4 [51], GLK1 [52] and CGA1 [53] (Supplementary Figure S4e). The low level of chlorophyll in prickles may result in reduced photosynthetic activity. This hypothesis is also supported by our study given that GO terms related to “photosynthesis” were enriched in the down-regulated genes in prickles. In conclusion, our morphological and transcriptome results suggest that the accumulation of flavonoids and the decrease in photosynthetic activity may occur simultaneously after the initiation of eggplants prickles.

Plant hormones play an important role in the development of plant organs. In the analysis of the enriched biological process, plant growth-related hormones (including auxin, cytokinin, ethylene and, gibberellin) and the stress-related hormones (including jasmonic acid and salicylic acid) were enriched (Supplementary Figure S4f, Supplementary Tables S5–S7). Our transcriptome data revealed that multiple hormone metabolism-related genes were either down- or up-regulated, indicating complex roles of plant hormones in prickle development. Among these hormones, cytokinin is known to have a positive effect on cell division [54,55]. One gene (Smechr1000130) encoding isopentenyl transferase (IPT), which is the first enzyme in the biosynthesis of cytokinin, was significantly up-regulated in developing prickles. Another gene (Smechr0402420) encoding cytokinin oxidase (CKX) involved in the degradation of cytokinin was up-regulated in matured prickles. Our results indicated that cytokinin is important in the initiation and development of prickles.

The molecular mechanisms underlying trichome morphogenesis have been extensively studied, and many key TFs responsible for trichome development have been identified in *Arabidopsis*, cotton, cucumber and tomato [15]. Similar as trichomes, prickles are also a protruding organ derived from the epidermis. We speculated that the molecular mechanisms observed in trichomes may also control prickle morphogenesis in eggplant. Comparative transcriptome studies of prickles in *S. viarum* and raspberry have shown that TFs such as MADS-box, MYB, AP2/ERF, and NAC might play a role in prickle development [10,11]. In this study, a total of 12 differentially expressed TFs potentially involved in prickle development were identified by homology search and expression analysis (Supplementary Table S10). The specific expression patterns of these TFs indicated their positive or negative role in prickle development. Of these genes, some are homologs of members of the WD-repeat/bHLH/MYB complex that controls trichome cell fate and patterning, and are highly expressed in developing prickles, such as bHLH family protein GL3 and WD repeat family protein TTG1 [56].The TFs identified in our study might be key regulators of prickle development in eggplant and merit further study.

Genetic analysis revealed that a major QTL located in chromosome 6 of eggplant is responsible for prickliness variability [13,14,44], and this locus was recently fine-mapped to a 133 kb interval [4]. Our transcriptome analysis indicated that four genes were significantly differentially expressed during prickle development, including three enzymes and one TF (Supplementary Table S11). Among these genes, Smechr0602826, which is homologous to AT4G30080, encodes an auxin response factor 16 (designated as *SmARF16* in our study). *AtARF16* has been reported to play a crucial role in regulating cell differentiation [57]. We found that *SmARF16* was highly expressed in developing prickles. Thus, *SmARF16* represents the strongest candidate gene responsible for prickle formation in eggplant. In the future, genetic transformation and phenotypic identification of this gene should be performed to further study its role in prickle development.

## 5. Conclusions

This work performed the phenotypic characterization and transcriptome analysis firstly on prickly and prickleless eggplant genotypes to understand prickle development at the morphological and molecular levels. Morphological analysis revealed that eggplant prickle was a multicellular, lignified, and layered organ. Comprehensive characterization of the expression profiles during prickle development identified key processes and hub genes involved in the cell cycle, flavonoid biosynthetic, photosynthetic and hormone metabolic processes. Furthermore, four genes were found to be differentially expressed within the *Pl* locus interval. Generally, this study identifies candidate genes responsible for prickle development, and provides new insights into the molecular pathways underlying prickle morphogenesis in eggplant. Additional studies are needed to functionally verify these candidate genes and investigate their role in prickle development. These genes could also be exploited in breeding programs to develop prickleless eggplant cultivars.

## Data Availability

The clean sequencing data in this study have been deposited in the NCBI Sequence Read Archive (SRA) under project PRJNA695792 (https://dataview.ncbi.nlm.nih.gov/object/PRJNA695792?reviewer=q7okd7fi1iasuq591dfsgmseb9 (accessed on 31 January 2021)).

## Supplementary Materials

The following are available online at https://www.mdpi.com/2073-4425/12/3/341/s1, Supplementary Table S1: Summary of sequenced RNA libraries, Supplementary Table S2: List of identified DEGs identified in prickleless epidermis vs. developing prickles comparison, Supplementary Table S3: List of identified DEGs in prickleless epidermis vs. matured prickles comparison, Supplementary Table S4: List of identified DEGs in developing prickles vs. matured prickles comparison, Supplementary Table S5: GO enrichment analysis of DEGs identified in the pairwise comparisons, Supplementary Table S6: GO enrichment analysis of up-regulated DEGs identified in the pairwise comparisons, Supplementary Table S7: GO enrichment analysis of down-regulated DEGs identified in the pairwise comparisons, Supplementary Table S8: K-means clusters, Supplementary Table S9: List of DEGs involved in development of eggplant prickles, Supplementary Table S10: Putative transcription factors involved in prickle development, Supplementary Table S11: List of DEGs within the *Pl* locus interval, Supplementary Table S12: Primers used for qRT-PCR, Supplementary Figure S1: Light micrographs of prickles and trichomes on the stem of prickly eggplant, Supplementary Figure S2: Heatmap of Pearson’s correlation coefficients between all pairs of samples, Supplementary Figure S3: GO enrichment analysis of DEGs identified in the pairwise comparisons, Supplementary Figure S4: Heatmap showing the expression patterns of the DEGs involved in biological processes during the development of prickles, Supplementary Figure S5: Heatmap showing the expression patterns of the differentially expressed TFs involved in the development of prickles, Supplementary Figure S6: Heatmap analysis of the genes within the *Pl* locus interval.
