# Supplementary material for: Comparative Transcriptome Analysis Reveals Key Genes and Pathways Involved in Prickle Development in Eggplant
Source: Genes (Basel). 2021 Feb 25;12(3):341. doi: 10.3390/genes12030341 (PMC7996550; doi:10.3390/genes12030341)
Supplement: Supplementary file 1 [file genes-12-00341-s001.zip › genes-1113945-supplementary/Supplementary Figures.docx]

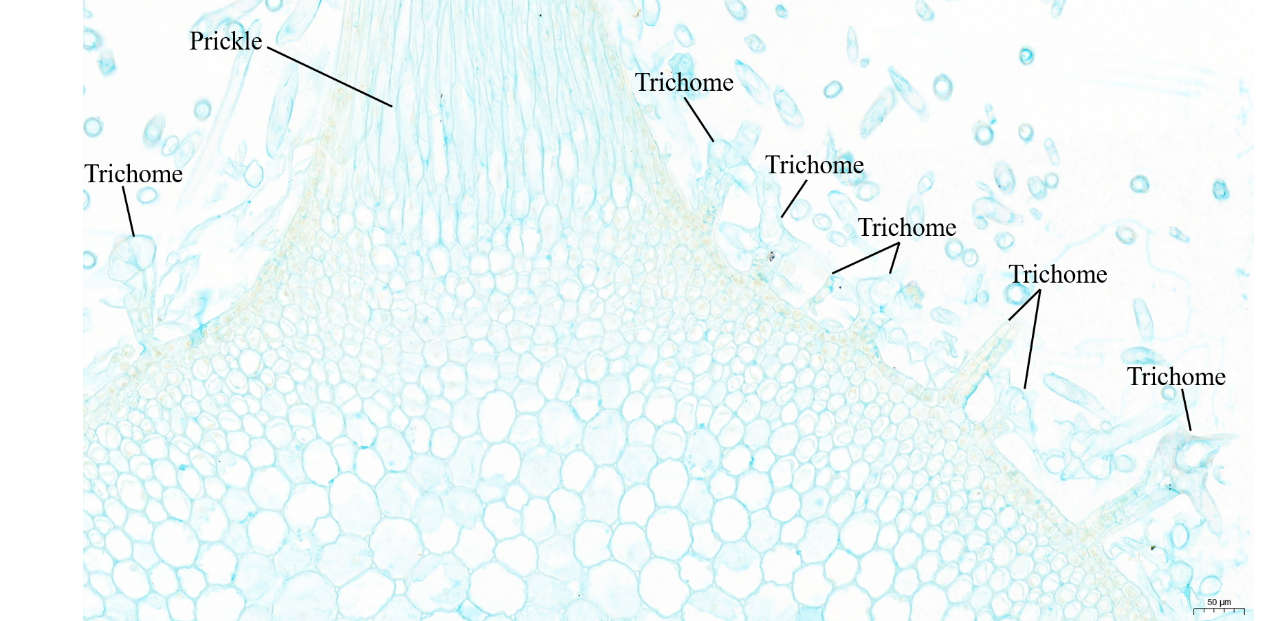


#### Supplementary Figure S1. Light micrographs of prickles and trichomes on the stem of prickly eggplant.


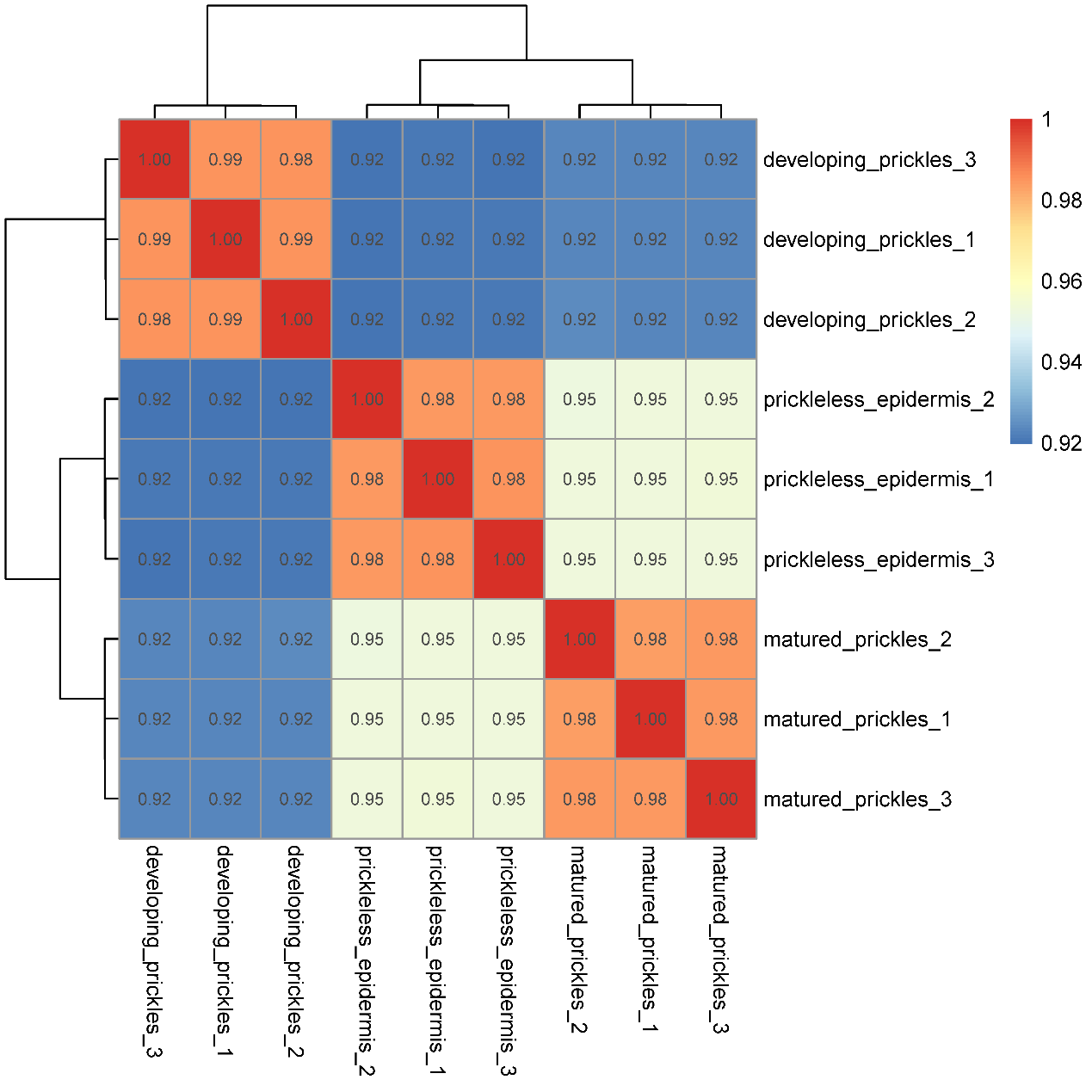


#### Supplementary Figure S2. Heatmap of Pearson's correlation coefficients between all pairs of samples.


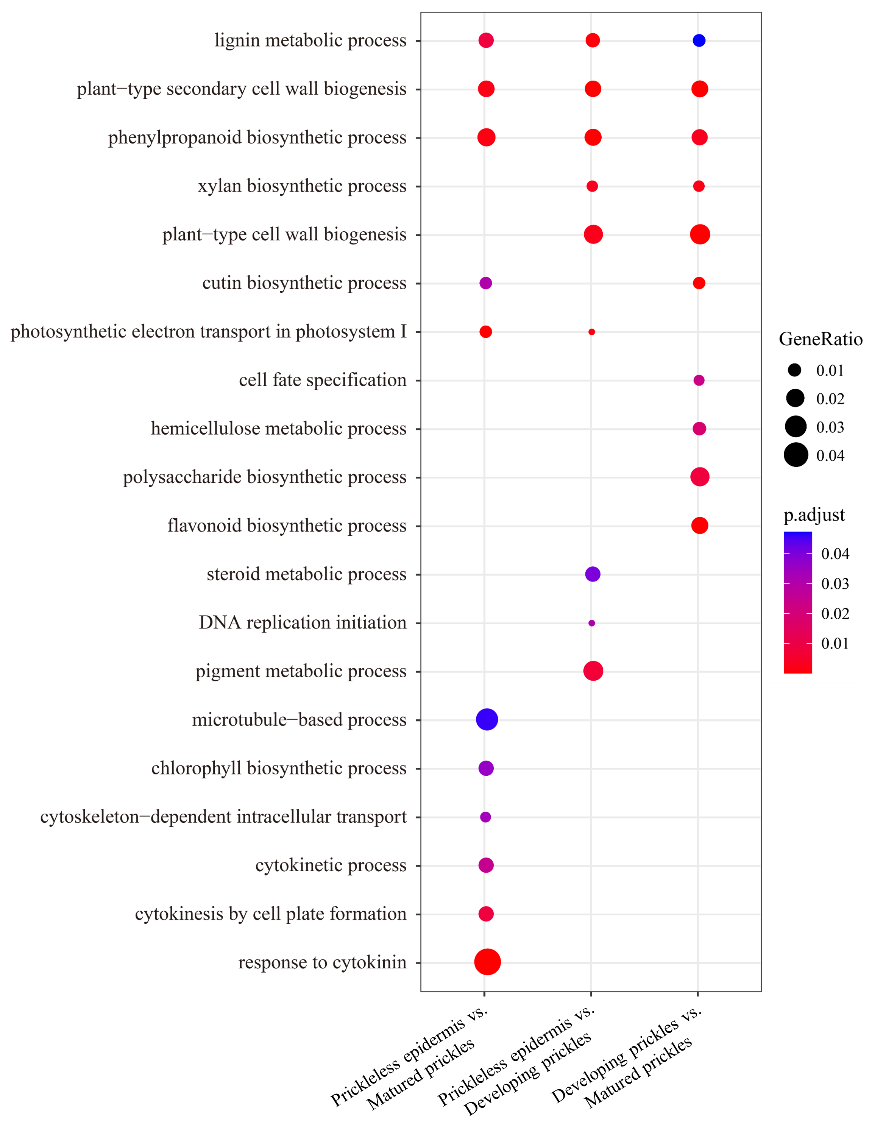


#### Supplementary Figure S3. GO enrichment analysis of DEGs identified in the pairwise comparisons.


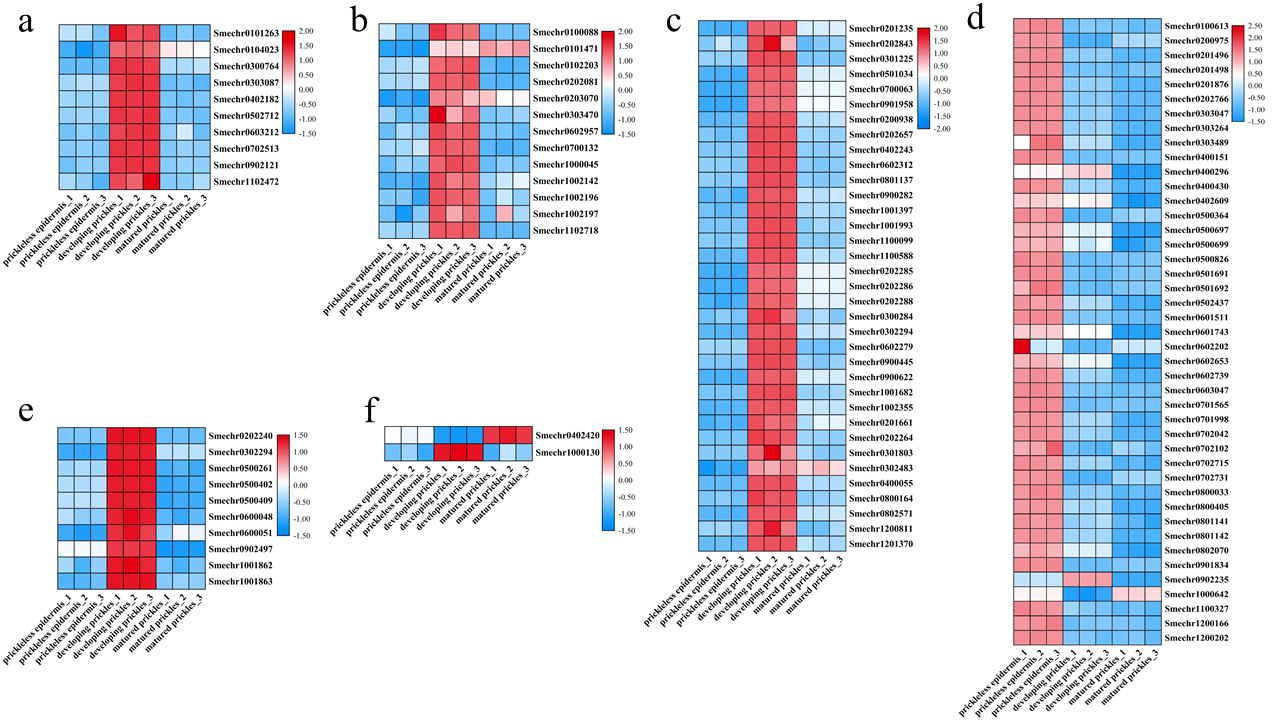


#### Supplementary Figure S4. Heatmap showing the expression patterns of the DEGs involved in biological processes during the development of prickles. (a) DEGs encoding key enzymes related to cytoskeleton and microtubule motor activity; (b) DEGs encoding key enzymes involved in DNA replication; (c) DEGs encoding key enzymes involved in cell wall biosynthesis; (d) DEGs encoding key enzymes involved in flavonoid biosynthesis; (e) DEGs encoding key enzymes involved in photosynthesis process; (f) DEGs encoding key enzymes involved in cytokinin signaling pathways. Heatmap was generated based on the normalized log_2_(TPM+1) values for each DEG. Blue and red scales represent relatively low or high expression, respectively.


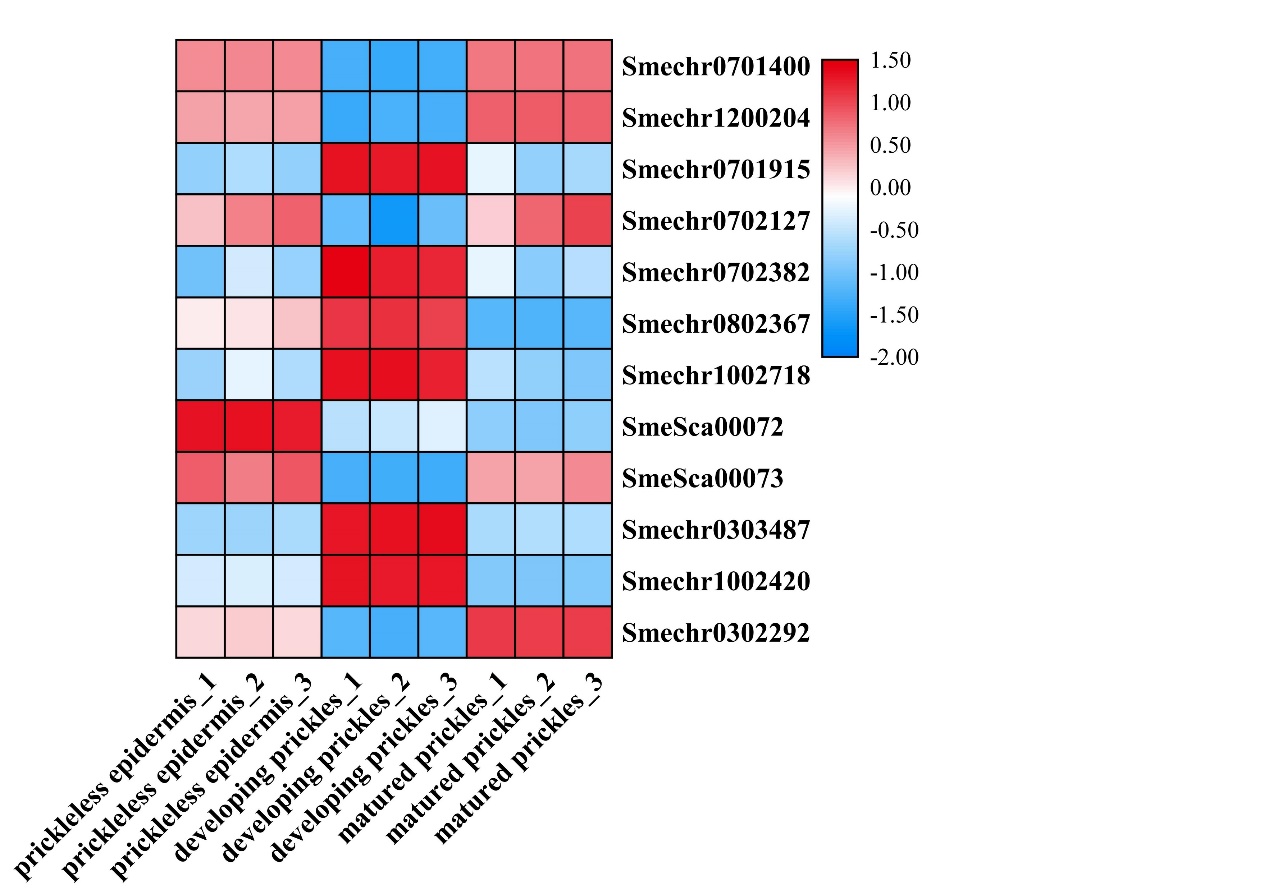


#### Supplementary Figure S5. Heatmap showing the expression patterns of the differentially expressed TFs involved in the development of prickles. Heatmap was generated based on the normalized log_2_(TPM+1) values for each DEG. Blue and red scales represent relatively low or high expression, respectively.


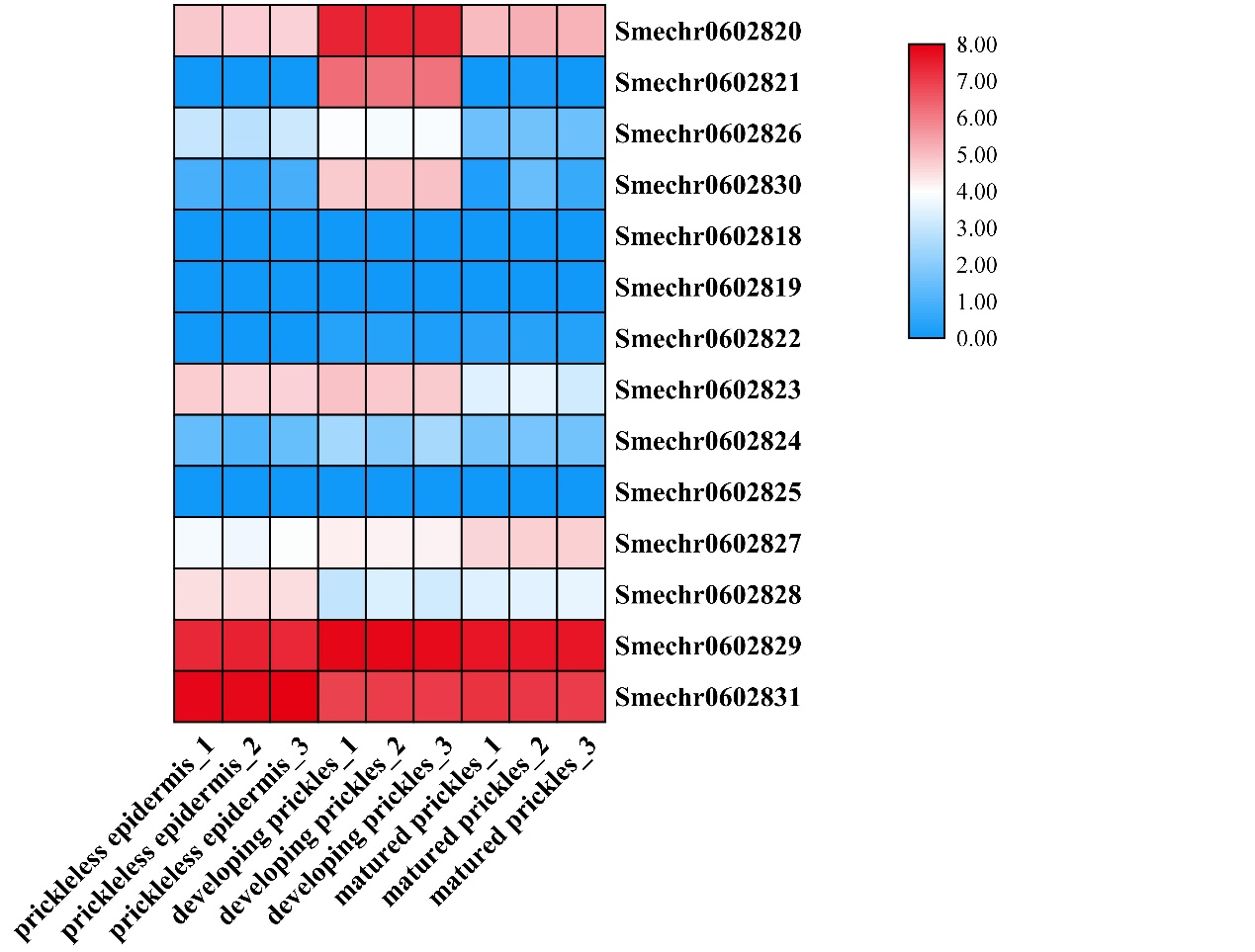


#### Supplementary Figure S6. Heatmap analysis of the genes within the *Pl* locus interval. Heatmap was generated based on the log_2_(TPM+1) values for each DEG. Blue and red scales represent relatively low or high expression, respectively.
